# Dataset and analysis of editorial board composition of 165 Hindawi journals indexed and abstracted in PubMed based on affiliations

**DOI:** 10.1016/j.dib.2018.05.065

**Published:** 2018-05-19

**Authors:** Hilary I. Okagbue, Aderemi A. Atayero, Muminu O. Adamu, Pelumi E. Oguntunde, Abiodun A. Opanuga, Sheila A. Bishop

**Affiliations:** a Department of Mathematics, Covenant University, Canaanland, Ota, Nigeria; b Department of Electrical and Information Engineering, Covenant University, Canaanland, Ota, Nigeria; c Department of Mathematics, University of Lagos, Akoka, Lagos, Nigeria

**Keywords:** Hindawi, Bibliometrics, Data analysis, PubMed, PubMed Central, Random, Smart campus, Ranking analytics, Statistics

## Abstract

This article explores the editorial board composition (across the six continents) of Hindawi journals indexed in PubMed. The dataset used is the official affiliation of the board members available at the various webpages of Hindawi journal website and not the countries of origin of the editorial board members. Summary statistics were presented and the raw dataset was provided for further analysis by interested scholars. The percentage of the editorial board composition across the continents was presented, the dataset of Hindawi journals indexed in both Hindawi and Scopus were also presented and measured in terms of Citescore and percentiles. The dataset can be used in journal evaluation, auditing, bibliometric analysis, management of smart campus; ranking and the analysis can be extended to other journal indexations.

**Specifications Table**

**Table:** 

Subject area	Decision Sciences
More specific subject area	Bibliometrics, Statistical data analysis
Type of data	Table, Figure and MS Excel
How data was acquired	The dataset was obtained freely from open access hindawi journals
Data format	Raw, partially analyzed
Experimental factors	Patterns of distribution of editorial members of journals indexed in PubMed.
Experimental features	Only the Journals indexed in PubMed were considered, journals indexed in both PubMed and Scopus but without Citescore and percentiles were excluded.
Data source location	Hindawi Publisher
Data accessibility	All the data are in this data article
Software	Excel, SPSS 21.0, Minitab 17.0.

**Value of the data**

• The dataset could be helpful in the evaluation of the impact of journal indexing on medical and other scientific publications.
• The data analysis can be extended to other reputable publishers.
• The dataset can be helpful in research output evaluation and auditing and in bibliometric analysis.
• The dataset can provide insight on the research volume of different continents and as such can be a criterion for ranking of journals and management of smart campuses.
• The research can be extended to include gender, population, education and development level gaps.
• The data analysis can be extended to capture the distribution of citations from the six continents and how it affects the editorial composition, manuscript acceptance and rejection.

## 1 Data

The dataset provided in this research relates to the editorial board composition of 165 Hindawi journals indexed in PubMed. It involves the official stated affiliations of the editorial board members grouped according to the continents namely; North America (NAM), Europe (EURO), Asia (ASIA), South America (SAM), Australia (AUST) and Africa (AFR). The grouping into continents was necessary because the data is large and highly skewed (some countries are not represented in the editorial board composition at all). The dataset was explored and the detailed summary is shown in Table 1. Also presented in this article are the impact of the journals indexed in both Scopus and PubMed measured in terms of their Citescore and percentiles.

**Table 1:** Summary statistics of the distribution of editorial board membership of PubMed indexed Hindawi journals across the six continents.

		NAM	EURO	ASIA	SAM	AUST	AFR
N	Valid	165	165	165	165	165	165
	Missing	0	0	0	0	0	0
Mean		14.77	22.64	8.88	1.28	1.42	0.52
Std. Error of Mean		2.754	4.410	2.151	0.351	0.282	0.128
Median		8.00	10.00	3.00	0.00	1.00	0.00
Mode		7	4	0^a^	0	0	0
Std. Deviation		35.379	56.642	27.633	4.514	3.619	1.644
Variance		1251.654	3208.341	763.566	20.376	13.098	2.702
Skewness		6.818	7.015	7.950	6.265	5.370	5.519
Std. Error of Skewness		0.189	0.189	0.189	0.189	0.189	0.189
Kurtosis		50.908	54.332	70.336	42.056	31.908	37.234
Std. Error of Kurtosis		0.376	0.376	0.376	0.376	0.376	0.376
Range		326	527	282	37	29	14
Minimum		1	1	0	0	0	0
Maximum		327	528	282	37	29	14
Sum		2437	3736	1466	212	234	86
Percentiles	25	5.00	6.00	1.00	.00	.00	.00
	50	8.00	10.00	3.00	.00	1.00	.00
	60	9.00	12.00	4.00	.00	1.00	.00
	75	13.00	18.00	7.00	1.00	1.00	.00
	90	17.40	41.00	17.00	2.40	3.00	1.00

a Multiple modes exist. The smallest value is shown

The raw dataset can be assessed as Supplementary data 1.

PubMed is a citation and abstract database and digital repository that archives and manages scholarly peer reviewed articles in the medical, biological, life and biochemical sciences. It is a bibliographic search engine used to access the Medical Literature Analysis and Retrieval System Online (MEDLINE). PubMed was released in 1996 and currently managed by the National Institute of Health (NIH) of the United States. It is closely related to PubMed Central managed by the National Center for Biotechnology Information (NCBI).

On the average, the editorial board composition across the continents can be interpreted using the inequality AFR < SAM < AUST < ASIA < NAM < EURO. Almost all Hindawi journals indexed in PubMed does not have editorial board members with affiliations in Africa, South America and Australia. The large variance for NAM, EURO and ASIA implies large deviation of the observations from the mean and consequently a high possibility that the editorial board composition may rise or fall below the mean. The high positive values of the skewness suggest that the data is highly right skewed; that is, there is high probability of observing low values. This is because there is uneven editorial composition across the 165 journals. It can also be seen that the high positive values of the Kurtosis implies that the probability of obtaining extreme values or values outside the range is high. All the journals have at least an editor whose affiliation is Europe or North America. Coefficient of variation can also be used to further explain the data.

The percentage of editorial board composition across the continents is presented in Table 2.

**Table 2:** Percentage and total number of editors across the continents. Remarks: 93.49% of the editorial board members have their affiliations domiciled in North America, Europe and Asia while the remaining percentage goes to the other three continents.

Continent	Total	Percentage
North America	2437	29.83
Europe	3736	45.72
Asia	1466	17.94
South America	212	2.60
Australia	234	2.86
Africa	86	1.05
Total	8171	100

## 2 Experimental design, materials and methods

The dataset is freely available at the various webpages of the publisher׳s website. The affiliations of the editorial board members were copied to Microsoft Excel, the countries of affiliations were matched with United Nations list of approved countries [1] and consequently classified according to their respective continents. Thereafter, statistical analyses were performed on the dataset.

The detailed statistical analysis of similar dataset can be found in [2], [3], [4], [5], [6], [7], [8], [9], [10], [11], [12], [13], [14], [15], [16], [17], [18], [19], [20], [21], [22], [23], [24], [25], [26], [27], [28], [29], [30], [31]. Chi-square test of goodness of fit was performed and shown in Table 3.

**Table 3:** Chi-square test of the editorial board composition across the continents.

	NAM	EURO	ASIA	SAM	AUST	AFR
Chi-Square	173.370	184.364	276.679	589.000	497.327	688.576
Df	30	44	28	9	11	7
Asymp. Sig.	0.000	0.000	0.000	0.000	0.000	0.000

The p-values indicated that the observed values differ greatly from the expected. This is to check if the expected number of editorial board composition is equivalent to the observed values. This is a kind of quality assurance.

Some of the journals are indexed in both PubMed and Scopus. In order to explore the relationship between the two indexations, the performance of the journals was explored using the Citescore and percentiles (performance metrics exclusive to Scopus). The journals indexed in Scopus without a Citescore and percentile were excluded and marked as missing values. The summary is shown in Table 4.

**Table 4:** Summary statistics for Citescore and Percentile of 97 Hindawi Journals indexed in PubMed and Scopus. Remarks: 97 out of 165 journals have both Citescore and percentiles. The average Citescore and percentile are 1.94 and 62 respectively; these show the high quality of Hindawi journals that are indexed in both Scopus and PubMed. The small values of the standard deviation indicate that most of the journals metrics clustered around the mean values. The analysis revealed that on the average, Hindawi journals indexed in Scopus and PubMed are Q2 journals and averagely cited as indicated by the distribution of the Citescore metric.

*Citescore*		*Percentile*	
Mean	1.943092784	Mean	62.81443299
Standard Error	0.082608275	Standard Error	1.666795306
Median	1.8	Median	64
Mode	1.8	Mode	64
Standard Deviation	0.813597158	Standard Deviation	16.41602996
Sample Variance	0.661940335	Sample Variance	269.4860395
Kurtosis	3.859655591	Kurtosis	−0.296157842
Skewness	1.466886445	Skewness	−0.3461562
Range	5.17	Range	75
Minimum	0.4	Minimum	22
Maximum	5.57	Maximum	97
Sum	188.48	Sum	6093
Count	97	Count	97
